# Supplementary material for: Defective homologous recombination DNA repair as therapeutic target in advanced chordoma
Source: Nat Commun. 2019 Apr 9;10:1635. doi: 10.1038/s41467-019-09633-9 (PMC6456501; doi:10.1038/s41467-019-09633-9)
Supplement: Supplementary file 1 — Supplementary Information [file 41467_2019_9633_MOESM1_ESM.pdf]

Supplementary Information

Defective homologous recombination DNA repair as therapeutic target in advanced chordoma

Gröschel, Hübschmann et al.

Supplementary Figures

Chord_01

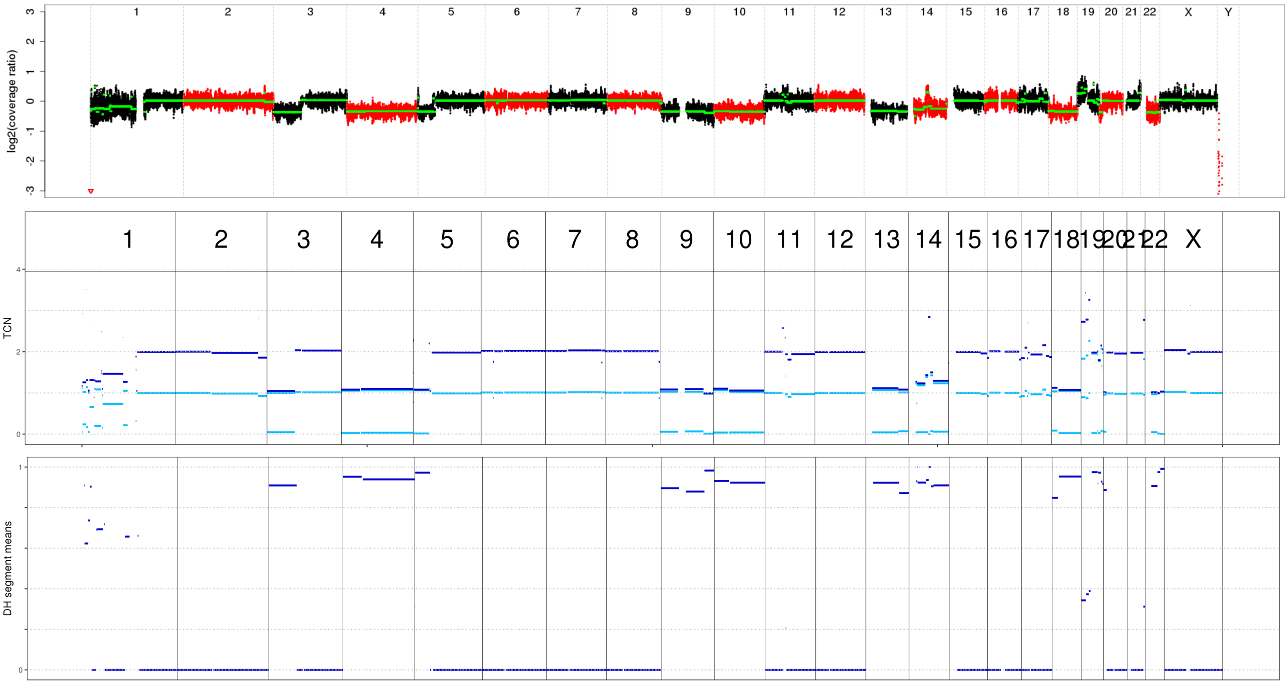

Chord_02

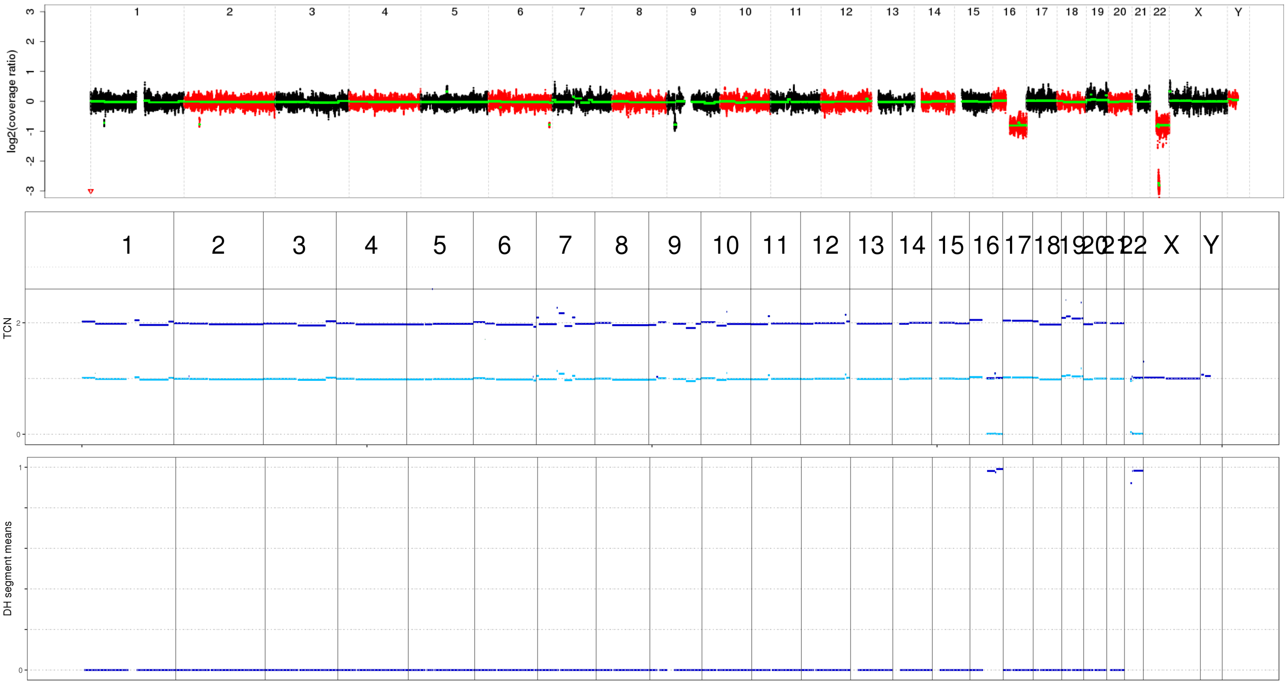

Chord_03

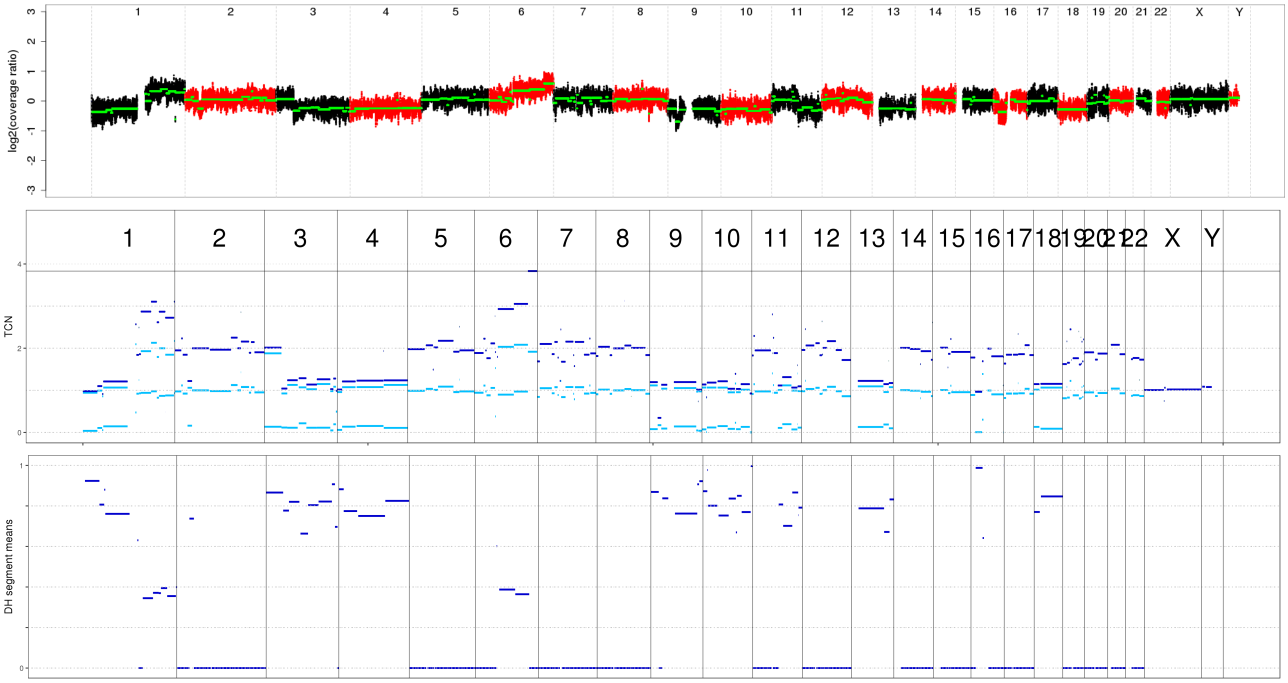

Chord_04

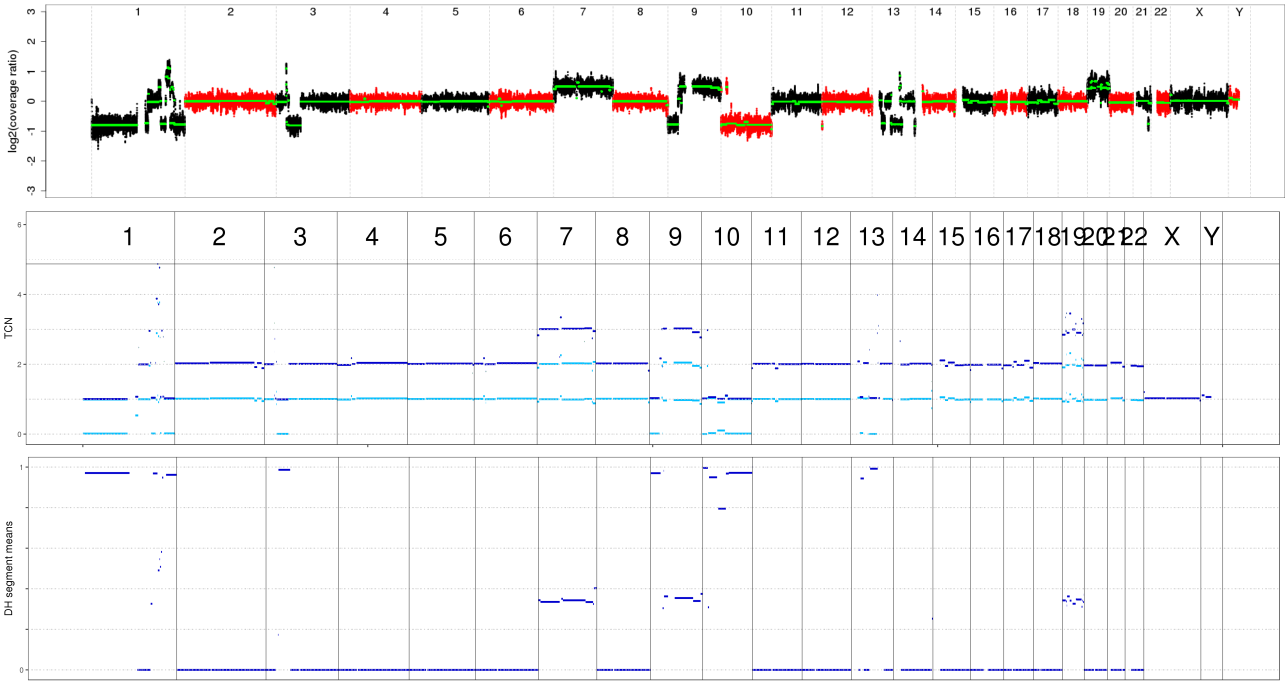

Chord_06

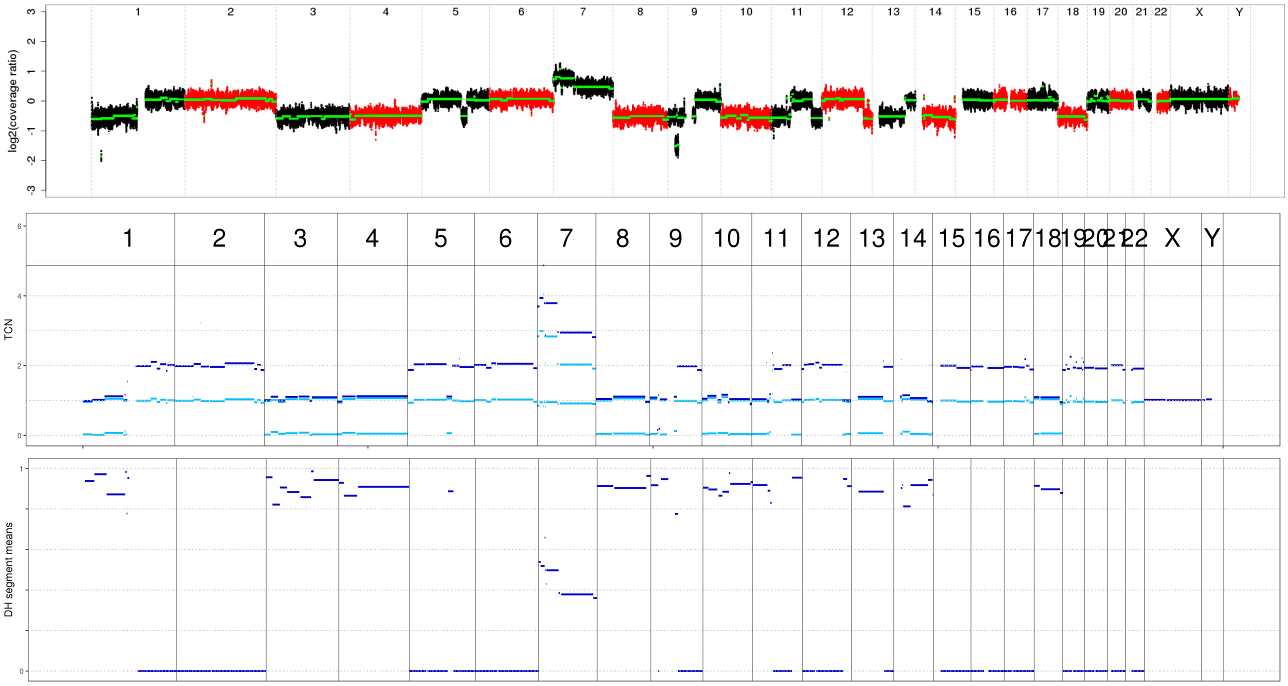

Chord_07

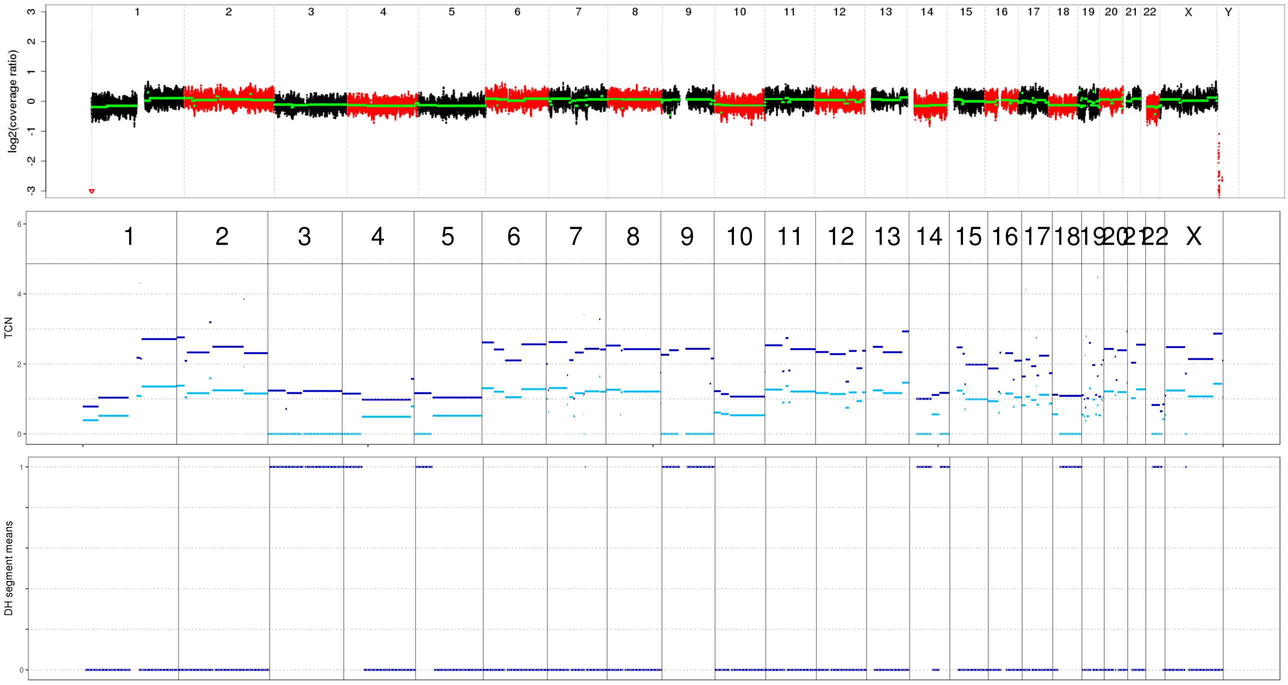

Chord_08

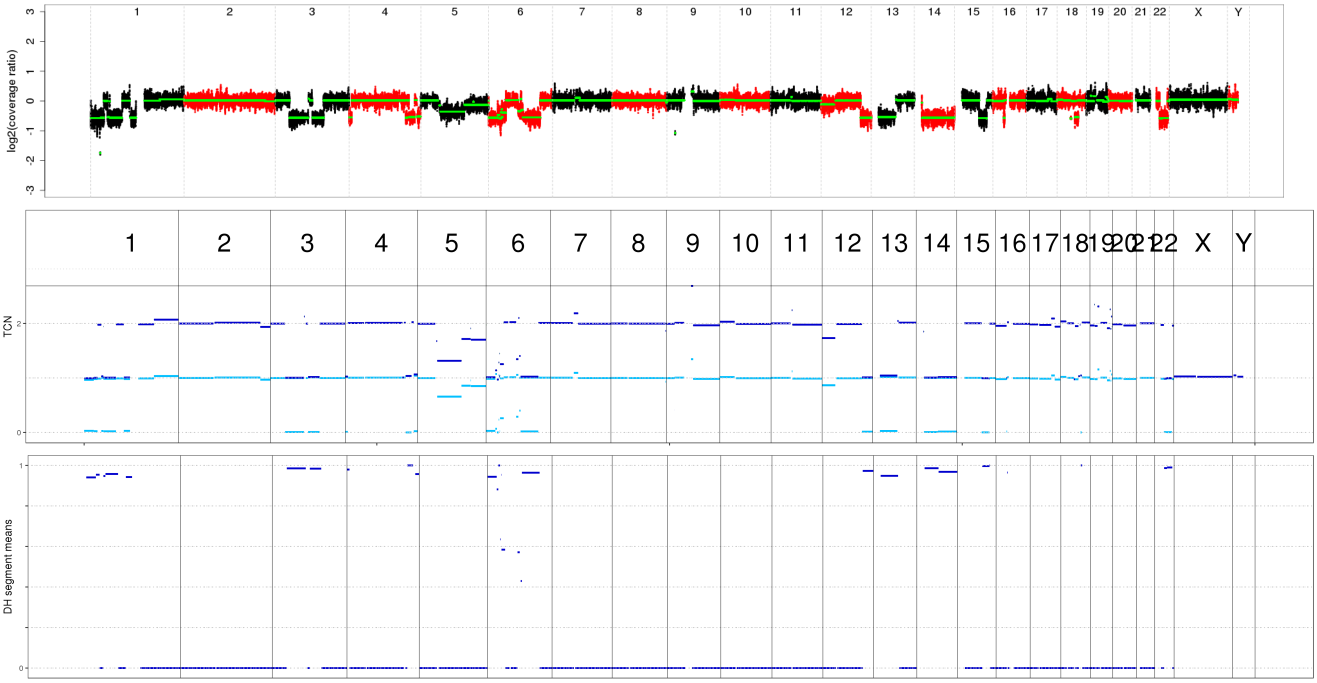

Chord_09

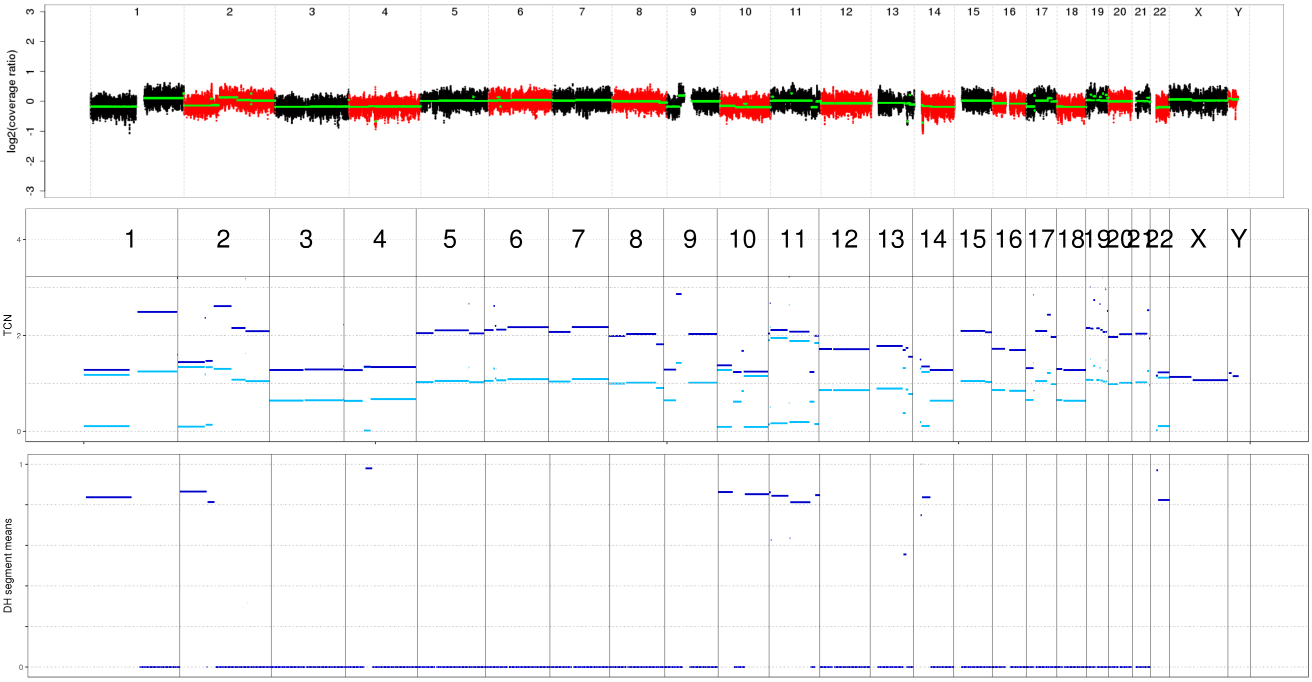

Supplementary Fig. 1 Copy number plots and CNA profiles of patients Chord_01, Chord_02, Chord_03, Chord_04, Chord_06, Chord_07, Chord_08, and Chord_09.

Chord_05R

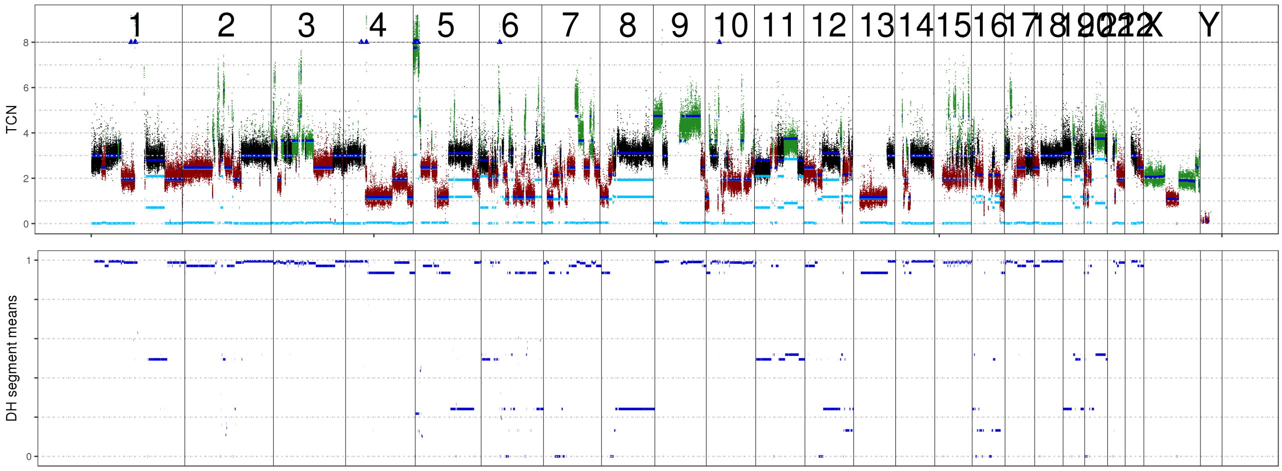

Chord_10

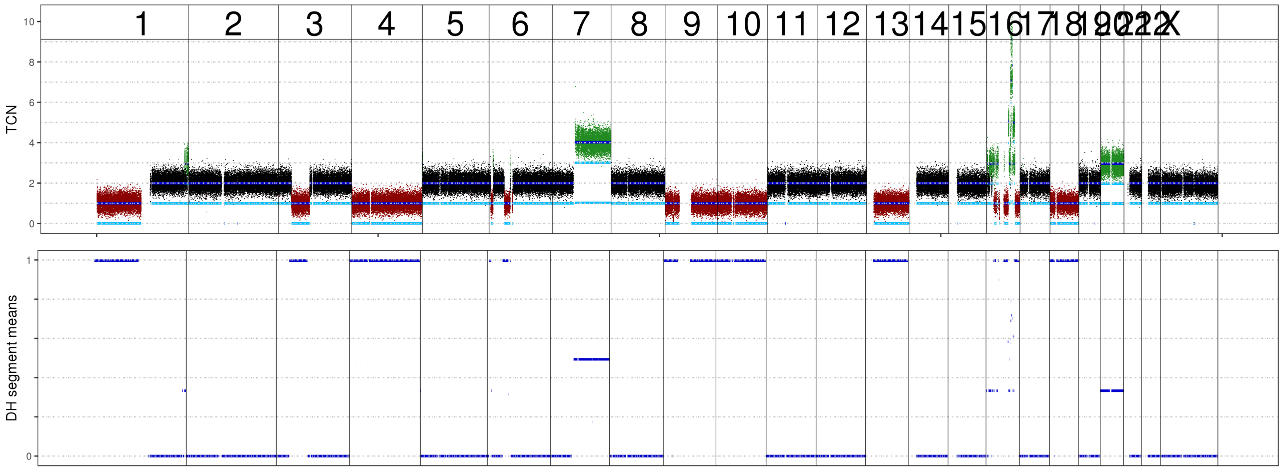

Chord_11

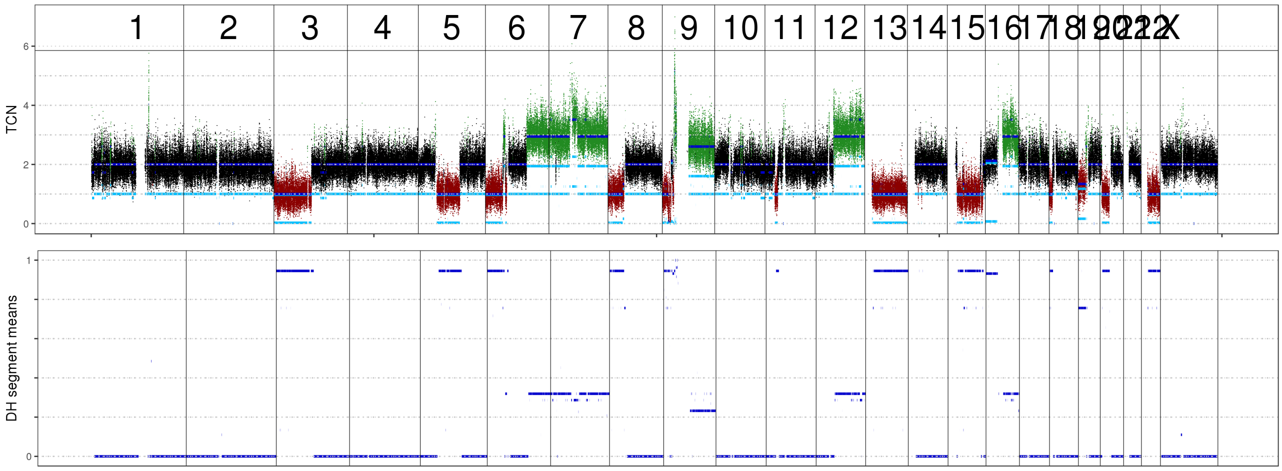

Supplementary Fig. 2 Copy number and allelic imbalance profiles of patients Chord_05 (at the time of acquired olaparib resistance), Chord_10, and Chord_11.

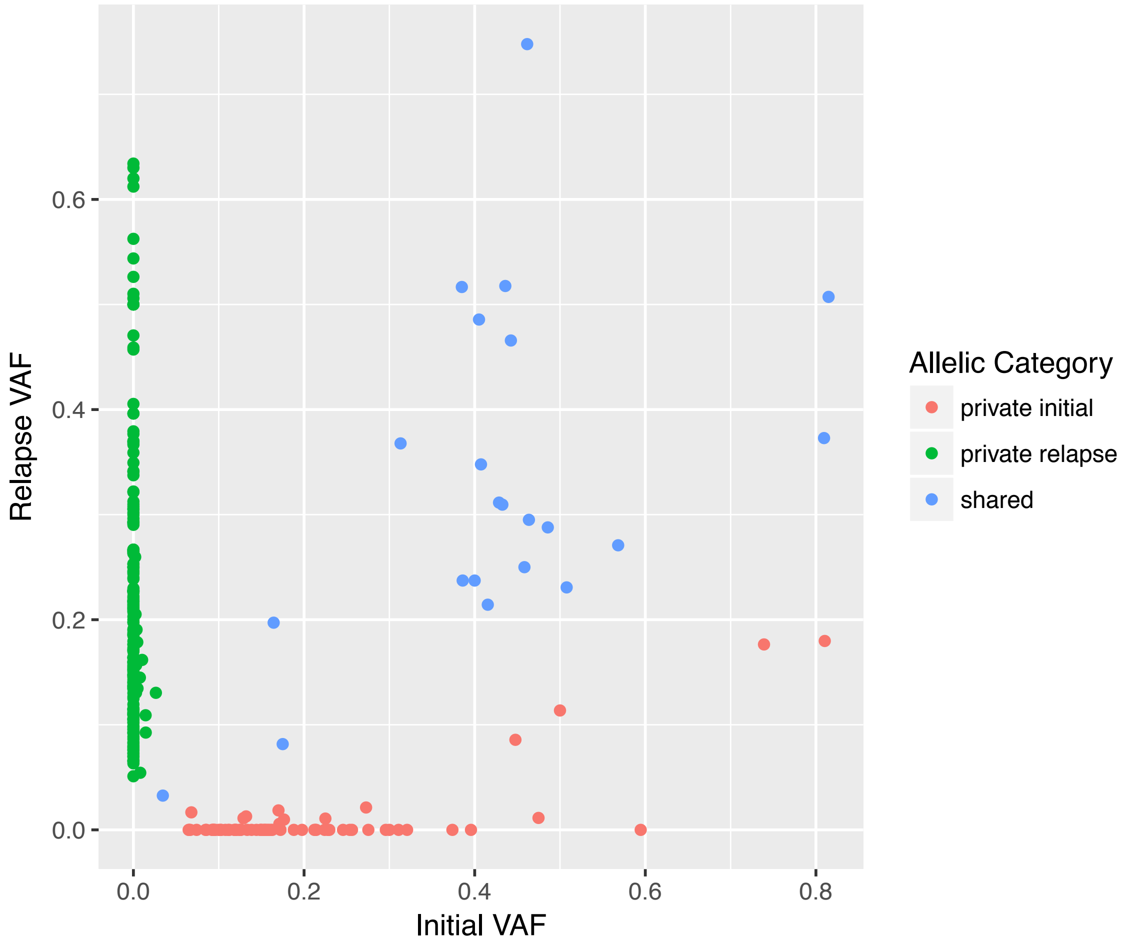

Supplementary Fig. 3 Variant allele fractions (VAF) of tumor specimens obtained from patient Chord_05 before ("Initial") and after ("Relapse") olaparib treatment. The underlying set of mutations consists of the merged variant calls from these two samples. Mutations were categorized based on their presence before ("private initial"), after ("private relapse"), or before and after ("shared") olaparib treatment.

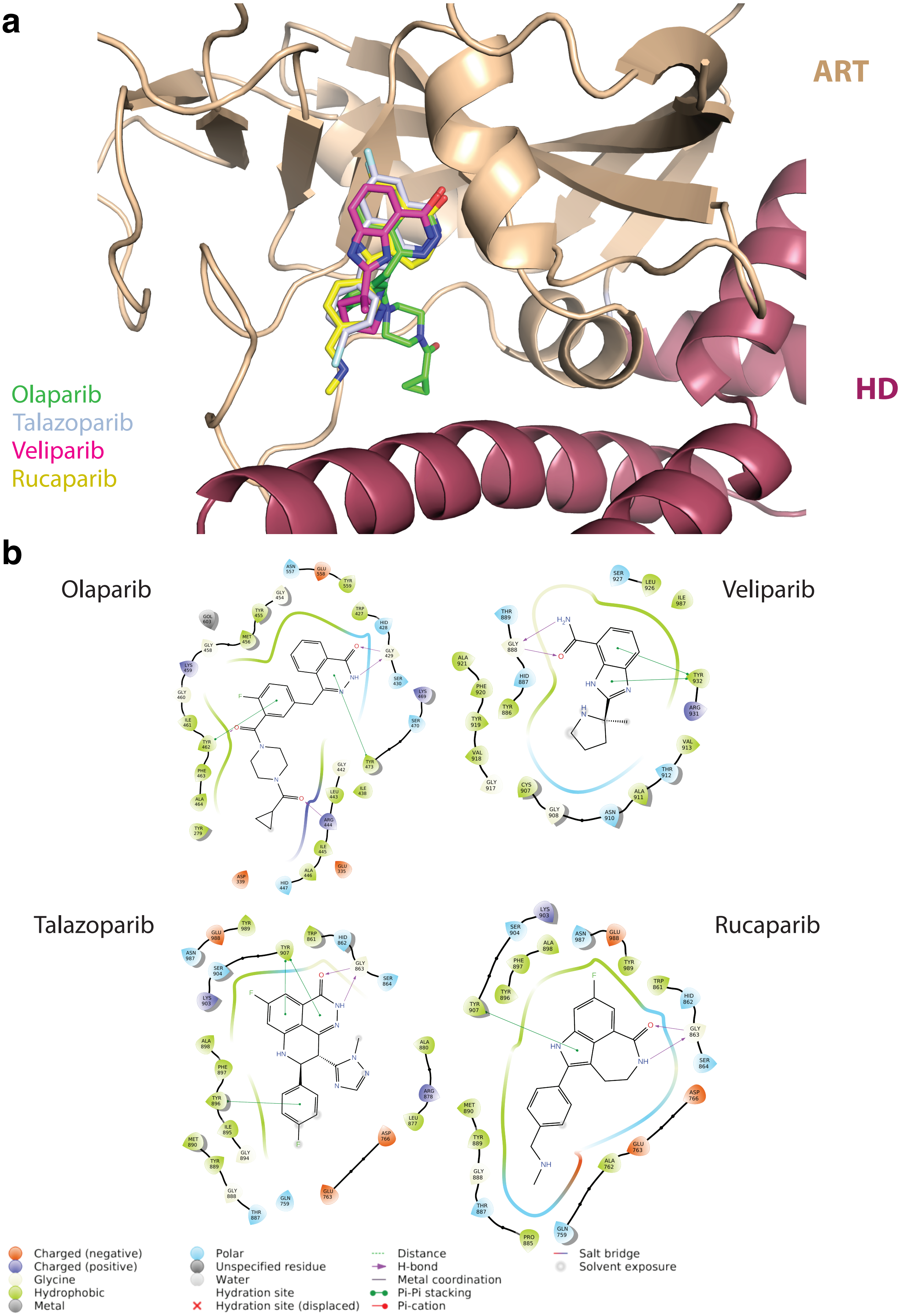
a
ART
HD
Olaparib
Talazoparib
Veliparib
Rucaparib
b
Olaparib
Veliparib
Talazoparib
Rucaparib
Charged (negative)
Charged (positive)
Glycine
Hydrophobic
Metal
Polar
Unspecified residue
Water
Hydration site
Hydration site (displaced)
Distance
H-bond
Metal coordination
Pi-Pi stacking
Pi-cation
Salt bridge
Solvent exposure

Supplementary Fig. 4 Structure comparison of four PARP1 inhibitors. **a** Superimposed view of olaparib (green; PDB ID: 4TVJ), talazoparib (blue and white; PDB ID: 4UND), veliparib (fuchsia; PDB ID: 5LX6), and rucaparib (yellow; PDB ID: 4RV6) obtained by structural alignment of the PARP1 catalytic site backbones of the four complex structures. The ART domain (PDB ID: 4UND) and the HD of PARP1 are depicted as wheat and raspberry cartoons, respectively. **b** Two-dimensional ligand interaction plots obtained for the four inhibitors using the Ligand Interaction Diagram function in Maestro (Schrödinger Release 2018-1: Maestro, Schrödinger, LLC, New York, NY, 2018).

Supplementary Tables

Supplementary Table 1 Patient characteristics and previous treatments

Sample	Age	Sex	Primary tumor site	Metastases	Previous treatment	Sequencing type
Chord_01	55	female	cervical	none	IMRT (70 Gy), surgery	WES
Chord_02	28	male	skull base	none	surgery, HIT (60.1 Gy)	WES
Chord_03	29	male	skull base	pulmonary, cerebellar (per continuity)	surgery, RT (45 Gy), imatinib	WES
Chord_04	55	male	cervical	none	surgery, RT (60 Gy)	WES
Chord_05	58	male	sacrum	none	PT (60 and 71.6 Gy), imatinib	WES
Chord_06	62	male	sacrum	lymphatic, bone, pulmonary	PT (74 Gy), surgery, RT (32 Gy), imatinib, sirolimus	WES
Chord_07	36	female	cervical	none	surgery, HIT (66 Gy), doxorubicin/ifosfamide	WES
Chord_08	40	male	cervical	none	surgery, RT (66 Gy)	WES
Chord_09	47	male	lumbar	pulmonary	surgery, RT (70 Gy), surgery, HIT (51 Gy), surgery	WES
Chord_10	72	female	sacrum	none	surgery, HIT (60 Gy), imatinib, sunitinib, imatinib/everolimus, erlotinib/bevacizumab	WGS
Chord_11	67	female	skull base	none	surgery, HIT (51 Gy), imatinib	WGS

IMRT, intensity-modulated radiotherapy; Gy, Gray; HIT, heavy ion therapy; RT, radiotherapy; PT, proton therapy

Supplementary Table 2 Sequencing coverages and additional quality parameters for WES samples

Sample	On-target ratio	Average sequencing coverage	Target capture (Agilent SureSelect)	Proper paired
Chord_01	74.21%	154.81	V5 without UTRs	99.22%
Chord_02	74.28%	163.76	V5 without UTRs	99.34%
Chord_03	63.82%	105.05	V5 with UTRs	98.51%
Chord_04	72.25%	119.47	V5 without UTRs	99.42%
Chord_05	75.07%	146.67	V5 with UTRs	98.69%
Chord_06	72.78%	111.10	V5 with UTRs	98.86%
Chord_07	75.38%	142.84	V5 with UTRs	99.96%
Chord_08	80.80%	198.17	V5 without UTRs	99.34%
Chord_09	76.33%	156.75	V5 with UTRs	99.12%

UTRs, untranslated regions

Supplementary Table 3 Sequencing coverages and additional quality parameters for WGS samples

Sample	Average sequencing coverage	Proper paired
Chord_05R	73.72	96.46%
Chord_10	76.09	96.24%
Chord_11	62.72	96.73%

Supplementary Table 4 Candidate HR genes and selected references

Gene	PubMed Identifier
ATM	26510020
BRCA1/2	26510020
CHEK2	26510020
ERCC3	26510020
FANCA	26510020
HDAC2	26510020
MLH3	26510020
MRE11	26510020
NBN	26510020
PALB2	26510020
RAD51	26510020
ATR	25965342, 27708213
ERCC6	25634215, 27374179
FANCC/D2/G	25609062, 28993682
PTEN	20049735, 20944090, 21468130, 23239809, 24625059
RAD18	25417706, 26056084
RAD51B	23239809, 24278037, 29465803
RAD54L	16912188, 26056084, 26669450, 28223274
RPA1	16912188, 23239809
XRCC3	17114795, 23512992, 23760496, 25028150, 29465803

Supplementary Table 5 Energy calculations for the *PARP1* p.T910A mutant

Domain	Total ddG
ART_LR	0.233
ART_HR	0.197
ART_HD_LR	1.865
ART_HD_HR	0.514

LR, Rosetta ddG_monomer protocol in low-resolution mode; HR, Rosetta ddG_monomer protocol in high-resolution mode; ART, constitutively active PARP1 structure (PDB ID: 5DS3); ART_HD, region corresponding to the ART domain and the HD (amino acids 662-1,011) of the PARP1-DNA complex structure (PDB ID: 4DQY)
