# Supplementary material for: Defective homologous recombination DNA repair as therapeutic target in advanced chordoma
Source: Nat Commun. 2019 Apr 9;10:1635. doi: 10.1038/s41467-019-09633-9 (PMC6456501; doi:10.1038/s41467-019-09633-9)
Supplement: Supplementary file 4 — Reporting Summary [file 41467_2019_9633_MOESM4_ESM.pdf]

nature research

Corresponding author(s): Stefan Gröschel and Stefan Fröhling

Last updated by author(s): Feb 11, 2019

Reporting Summary

Nature Research wishes to improve the reproducibility of the work that we publish. This form provides structure for consistency and transparency in reporting. For further information on Nature Research policies, see Authors & Referees and the Editorial Policy Checklist.

Statistics

For all statistical analyses, confirm that the following items are present in the figure legend, table legend, main text, or Methods section.

n/a	Confirmed	
☐	☒	The exact sample size (*n*) for each experimental group/condition, given as a discrete number and unit of measurement
☐	☒	A statement on whether measurements were taken from distinct samples or whether the same sample was measured repeatedly
☐	☒	The statistical test(s) used AND whether they are one- or two-sided<br>*Only common tests should be described solely by name; describe more complex techniques in the Methods section.*
☒	☐	A description of all covariates tested
☒	☐	A description of any assumptions or corrections, such as tests of normality and adjustment for multiple comparisons
☐	☒	A full description of the statistical parameters including central tendency (e.g. means) or other basic estimates (e.g. regression coefficient) AND variation (e.g. standard deviation) or associated estimates of uncertainty (e.g. confidence intervals)
☐	☒	For null hypothesis testing, the test statistic (e.g. *F*, *t*, *r*) with confidence intervals, effect sizes, degrees of freedom and *P* value noted<br>*Give P values as exact values whenever suitable.*
☒	☐	For Bayesian analysis, information on the choice of priors and Markov chain Monte Carlo settings
☒	☐	For hierarchical and complex designs, identification of the appropriate level for tests and full reporting of outcomes
☒	☐	Estimates of effect sizes (e.g. Cohen's *d*, Pearson's *r*), indicating how they were calculated

Our web collection on statistics for biologists contains articles on many of the points above.

Software and code

Policy information about availability of computer code

Data collection	All resources used are described in the Methods section. Publicly available software included the human reference genome (build 37, version hs37d5), ExAC (v.0.3.1), GENCODE (v19), dbSNP (build 141), 1000 Genomes Project, PDB, Rosetta, and PyMOL (v1.7.x).
Data analysis	All software used is described in the Methods section. Publicly available software included bwa-mem (version 0.7.15), biobambam (version 0.0.148), Sambamba (version 0.6.5), samtools, Platypus (version 0.8.1), R, ANNOVAR, ACEseq, YAPSA, Picard, bedtools (v2.25.0), segemehl (v0.2.0), and cnvKit (version 0.9.3).

For manuscripts utilizing custom algorithms or software that are central to the research but not yet described in published literature, software must be made available to editors/reviewers. We strongly encourage code deposition in a community repository (e.g. GitHub). See the Nature Research guidelines for submitting code & software for further information.

Data

Policy information about availability of data

All manuscripts must include a data availability statement. This statement should provide the following information, where applicable:

- Accession codes, unique identifiers, or web links for publicly available datasets
- A list of figures that have associated raw data
- A description of any restrictions on data availability

Sequencing data were deposited in the European Genome-phenome Archive under accession EGAS00001002720.

Field-specific reporting

Please select the one below that is the best fit for your research. If you are not sure, read the appropriate sections before making your selection.

☒ Life sciences ☐ Behavioural & social sciences ☐ Ecological, evolutionary & environmental sciences

For a reference copy of the document with all sections, see nature.com/documents/nr-reporting-summary-flat.pdf

Life sciences study design

All studies must disclose on these points even when the disclosure is negative.

Sample size	Sample size was determined by tumor/control samples available.
Data exclusions	No data were excluded.
Replication	Whole-genome sequencing (WGS) and whole-exome sequencing (WES) were performed once per sample (typical convention in cancer genome sequencing and personalized oncology).
Randomization	No randomization was performed
Blinding	Investigators were not blinded to allocation.

Reporting for specific materials, systems and methods

We require information from authors about some types of materials, experimental systems and methods used in many studies. Here, indicate whether each material, system or method listed is relevant to your study. If you are not sure if a list item applies to your research, read the appropriate section before selecting a response.

Materials & experimental systems

n/a	Involved in the study	
☒	☐	Antibodies
☒	☐	Eukaryotic cell lines
☒	☐	Palaeontology
☒	☐	Animals and other organisms
☐	☒	Human research participants
☐	☒	Clinical data

Methods

n/a	Involved in the study	
☒	☐	ChIP-seq
☒	☐	Flow cytometry
☒	☐	MRI-based neuroimaging

Human research participants

Policy information about studies involving human research participants

Population characteristics	We performed WES (n = 9) and WGS (n = 2) of tumor tissue and matched blood from 11 patients (age, 27-72 years) with locally advanced and/or metastatic chordoma who were enrolled in the MASTER (Molecularly Aided Stratification for Tumor Eradication Research) program, a registry trial for younger adults with advanced cancer across all histologies and patients with rare tumors. All patients had previously received radiotherapy to the primary tumor site, following surgical resection in nine of 11 cases. Systemic treatment had been administered in six of 11 cases. All patients had progressive disease prior to molecular analysis. Detailed clinical information is provided in Supplementary Table 1.
Recruitment	For WES and WGS, fresh-frozen tumor specimens and matched normal control samples were collected from adult patients who had been diagnosed with chordoma according to World Health Organization criteria at four German cancer centers (NCT Heidelberg and Heidelberg University Hospital, Heidelberg; West German Cancer Center, Essen; University Hospital Carl Gustav Carus, Dresden; Frankfurt University Hospital, Frankfurt). Samples were pseudonymized, and tumor histology and cellularity were assessed at the Institute of Pathology, Heidelberg University Hospital, prior to further processing.
Ethics oversight	All patients provided written informed consent under a protocol approved by the Ethics Committee of Heidelberg University, and the study was conducted in accordance with the Declaration of Helsinki.

Note that full information on the approval of the study protocol must also be provided in the manuscript.

Clinical data

Policy information about clinical studies

All manuscripts should comply with the ICMJE guidelines for publication of clinical research and a completed CONSORT checklist must be included with all submissions.

Clinical trial registration	*Provide the trial registration number from ClinicalTrials.gov or an equivalent agency.*
Study protocol	*Note where the full trial protocol can be accessed OR if not available, explain why.*
Data collection	*Describe the settings and locales of data collection, noting the time periods of recruitment and data collection.*
Outcomes	*Describe how you pre-defined primary and secondary outcome measures and how you assessed these measures.*
